# A Capability Approach to worker dignity under Algorithmic Management

**DOI:** 10.1007/s10676-022-09637-y

**Published:** 2022-02-03

**Authors:** Laura Lamers, Jeroen Meijerink, Giedo Jansen, Mieke Boon

**Affiliations:** 1 Department of Industrial Engineering and Business Information Systems, Human Resource Management Research Group, University of Twente, Enschede, Netherlands; 2 Department of Public Administration, University of Twente, Enschede, Netherlands; 3 Department of Philosophy, University of Twente, Enschede, Netherlands

**Keywords:** Algorithmic Management, Capability Approach, Human Resource Management, Worker dignity

## Abstract

This paper proposes a conceptual framework to study and evaluate the impact of ‘Algorithmic Management’ (AM) on worker dignity. While the literature on AM addresses many concerns that relate to the dignity of workers, a shared understanding of what worker dignity means, and a framework to study it, in the context of software algorithms at work is lacking. We advance a conceptual framework based on a Capability Approach (CA) as a route to understanding worker dignity under AM. This paper contributes to the existing AM literature which currently is mainly focused on exploitation and violations of dignity and its protection. By using a CA, we expand this focus and can evaluate the possibility that AM might also enable and promote dignity. We conclude that our CA-based conceptual framework provides a valuable means to study AM and then discuss avenues for future research into the complex relationship between worker dignity and AM systems.

## Introduction

In recent years, there has been a rapid growth in the use of software algorithms to automate Human Resource Management (HRM) practices (Cheng & Hackett, 2021). This increasing use of algorithmic technology to manage workforces is known in the academic literature as ‘Algorithmic Management’ (Duggan et al., 2020; Lee et al., 2015; Leicht-Deobald et al., 2019; Meijerink & Bondarouk, 2021; Möhlmann & Zalmanson, 2017). Algorithmic Management (hereafter: AM) can be understood as an umbrella term that refers to data-driven systems in which software algorithms (semi-) automate and execute HRM-related decision-making that affects workers. The character of AM changes the practice of HRM from a human- to a technology-driven process (Schildt, 2017). Today, many HRM practices traditionally performed by middle or lower management are automated and executed by algorithmic systems (Cherry & Aloisi, 2016; Duggan et al., 2020; Meijerink & Bondarouk, 2021; Möhlmann & Zalmanson, 2017).

Automating HRM tasks using algorithms has proven to be highly profitable for companies as limiting human interaction improves decision-making efficiency (Jarrahi et al., 2019; Möhlmann & Zalmanson, 2017; Walker et al., 2021) and enables organisations to coordinate and evaluate workers on a large scale (Kellogg et al., 2020). A wide range of HRM-related practices and decisions are supported or taken over by algorithmic systems including *staffing* in terms of automated resume screening (Cheng & Hackett, 2021; Leicht-Deobald et al., 2019), *matching* by automatically assigning workers to tasks (Rosenblat & Stark, 2016) and algorithmically integrating data-based performance measures for *evaluation*, *appraisal* and *compensation* purposes (Jarrahi et al., 2019; Kellogg et al., 2020).

The use of AM affects workers, and scholars have started to critically study the effects of AM on the working life. A growing concern in this field of study is that AM comes at the expense of worker interests. More specific and rather alarming concerns are linked to the instrumentalisation and dehumanisation of work(ers) that AM precipitates (De Stefano, 2020; Gal et al., 2020; Gandini, 2019; Kellogg et al., 2020; Meijerink & Bondarouk, 2021; Veen et al., 2020). For example, Gandini (2019**)** addresses how algorithmic rating systems turn real-world experiences into numbers or stars, and quantify the workers subject to this system, thereby dehumanising them. Moreover, there are many articles that show how AM instrumentalises workers through soft surveillance and by gaining economic value out of workforces (Kellogg et al., 2020; Newlands, 2020; Veen et al., 2020).

Through dehumanisation and instrumentalisation, AM is considered to violate the inherent dignity of workers, which is why scholars have called for the protection of worker dignity under AM (De Stefano, 2020; Rosenblat et al., 2017). Although the perspective on the violation of inherent dignity is important, the current academic debate overlooks the possibilities that AM could promote dignity. This is surprising given that information systems studies have shown that information technologies can both enable and restrain desired outcomes (Bondarouk et al., 2017; Meijerink & Bondarouk, 2021; Orlikowski, 1992). Moreover, dignity research shows that, rather than being merely inherent, dignity can also be contingent—i.e., a human worth that is earned and, thus, to be promoted (e.g. Bal, 2017; Pirson et al., 2016). To advance the field further, we see a need for a conceptual framework for worker dignity under AM that allows us to study and evaluate both the *protection of inherent dignity* as well as the *promotion of contingent dignity*.

This paper advances such a conceptual framework by drawing on the Capability Approach (CA). The CA, initially advocated by Amartya Sen (e.g. 1992; 1999) and later developed by Martha Nussbaum (e.g. 2000; 2006; 2011), is a normative framework that is used to evaluate individual well-being and development, and also higher-level aspects of social arrangements, policies and social change (Robeyns, 2005). With the value of human dignity at its heart, the CA discourages looking at income, resources, primary goods, utility or preference satisfaction to evaluate human development (ibid). Rather, CA advocates focus on human capabilities, best described in this context as the opportunities for that what people are effectively able to do and be – for example, the opportunity for living healthily or being able to learn.

An essential aspect of the CA is that developing the capabilities to live a life worthy of living adds to an agent’s dignity (Robeyns, 2005). As such, the CA is essentially a dignity-centred normative framework that recognises both inherent and contingent dignity. On the one hand, the CA acknowledges the Kantian idea of inherent dignity by recognising human value as an inherent worth and going beyond instrumentalist paradigms that lead to evaluating development in monetary terms. On the other hand, the CA also respects the Aristotelean idea of contingent dignity as something that can be earned by adopting an agent-based approach that starts from individual conceptions of what a dignified life is. The CA thus allows study of dignity promotion while not overlooking dignity violation. Accordingly, this paper presents a CA-inspired conceptual framework for both dignity violation and dignity promotion in a context where software algorithms (semi-)automate HRM activities.

Our paper is structured as follows. We begin by explaining AM and demarcating the AM debate with a focus on its impacts on workers. We continue by discussing inherent dignity and contingent dignity as two relevant interpretations of dignity for management research. This is followed by outlining how the CA combines these interpretations and a discussion on how the CA can help when studying AM. We conclude by reflecting on the implications of the CA for future AM research.

## Algorithmic Management and its impacts on workers

The ‘Algorithmic Management’ (AM) concept was first coined by Lee et al. (2015) to describe how Uber’s software algorithms allow workers to be “assigned, optimised, and evaluated through algorithms and tracked data.” (p. 1603). It has since been used to address various developments linked to automation and uses of software algorithms in HRM processes. The automation of HRM practices is the most frequently discussed in the platform economy context, where digital labour platforms such as Fiverr, Deliveroo and Upwork found highly efficient ways to coordinate large workforces (Jarrahi et al., 2019). Although AM was initially and most extensively adopted by platforms (Jarrahi et al., 2019), it is increasingly adopted outside the platform economy (Cheng & Hackett, 2021; Jarrahi et al., 2021) and comes in many forms, with various levels of human involvement (Leicht-Deobald et al., 2019) in directing, evaluating and disciplining workers (Kellogg et al., 2020).

AM, in terms of controlling workers on a large scale by automated decision-making with limited human intervention, has proven highly profitable for companies (Jarrahi et al., 2019; Möhlmann & Zalmanson, 2017). However, it is increasingly argued that it comes at the expense of workers’ interests. Various reports and articles refer to the adverse effects of AM, such as limiting sensemaking among workers (Jarrahi et al., 2019) and detrimental effects of management-by-algorithms on worker autonomy (Möhlmann & Zalmanson, 2017; Rosenblat, 2018; Shapiro, 2018), personal integrity (Leicht-Deobald et al., 2019), job-quality (Veen et al., 2019). Although addressing distinct concepts, these studies coalesce in highlighting implications of AM that centre around the *violation* of *human dignity* in terms of *dehumanisation* and *instrumentalisation*.

To address why and how dehumanisation and instrumentalisation arise under AM, we need to consider how AM is explained in the debate and what scholars have argued to be its characteristics. In the literature, AM has been defined in various ways. Prominent notions are, for instance, that of Duggan et al. (2020), who described it as: “a system of control where self-learning algorithms are given the responsibility for making and executing decisions affecting labour, thereby limiting human involvement and oversight of the labour process.” (p. 119). Another much-cited definition is offered by Lee et al. (2015), who “call software algorithms that assume managerial functions and surrounding institutional devices that support algorithms in practice, algorithmic management.” (p. 1603). According to Schildt (2017), such “algorithms track the performance of employees or contractors, optimising decisions concerning their tasks and future employment” (p. 25). Similarly, Gal et al. (2020) explain AM as “computational techniques that leverage digital data from multiple organisational areas to reflect different facets of members’ behaviour” (p. 9). Notwithstanding differences (e.g. regarding the self-learning nature of algorithms, the involvement of humans and the type of managerial responsibilities), these definitions come together in outlining three key characteristics of AM: datafication/quantification, automation and optimisation. Acknowledging these characteristics, it becomes evident that the dignity of workers is at stake when interacting with AM. We now discuss the characteristics and their possible implications for human dignity.

An initial characteristic of AM is that it triggers the *datafication* and *quantification* of work since AM systems are data-driven (Newlands, 2020; Strohmeier, 2020). Generally, AM functions as a system with input, throughput and output (Meijerink & Bondarouk, 2021). Critical to algorithmic systems is that the input is machine-readable data (e.g. Newlands, 2020), which means that human experiences and real-life situations are converted into machine-comprehensible datasets. The input to AM systems are data acquired from workers’ mobile applications and devices (Strohmeier, 2020). The data regarding worker behaviour, traits, moods or location, together with a set of rules (software codes), enable automated processing, which can be understood as the throughput of the system (Garcia-Arroyo & Osca, 2019; Strohmeier & Piazza, 2015). Eventually, this leads to a system output comprising decisions concerning managerial practices that are similarly articulated in quantified terms (Rosenblat & Stark, 2016). An additional problem of such data-driven management is that AM systems are opaque, leading to information asymmetries between the worker and the organization (Cheng & Foley, 2019; Jarrahi et al., 2021; Rosenblat & Stark, 2016). More generally, research on the use of big data has shown severe biases in data-driven systems, a problem that also holds for AM (e.g. Lee, 2018).

The second AM characteristic is the *automation* of HRM activities. Automation comes in various degrees as AM systems differ regarding the decision-making power that is granted to the algorithms (Leicht-Deobald et al., 2019; Meijerink et al., 2021). This decision-making power may range from semi-automation (where algorithms augment decision-making by HR managers by offering information and insight) through to full automation (where decision-making power has shifted strongly or solely towards the algorithms). Not only the level of automation but also the types of HRM practices that are automated by software algorithms vary. The range of automated HRM practices is broad and stretches into several functional HRM areas (Meijerink & Bondarouk, 2021). HRM practices that were traditionally performed by middle management but are now found under the umbrella of AM include *staffing* in terms of resumé screening by a computer; *training* based on the algorithmic prediction of skill gaps; *appraisal* based on big data analysis of worker performance; *workforce planning*, such as algorithmically assigning workers to shifts or jobs (also referred to as matching or matchmaking), and *compensation and reward* (Cheng & Hackett, 2021; Kellogg et al., 2020; Leicht-Deobald et al., 2019; Newlands, 2020; Strohmeier & Piazza, 2015).^1^

**Fig. 1:**
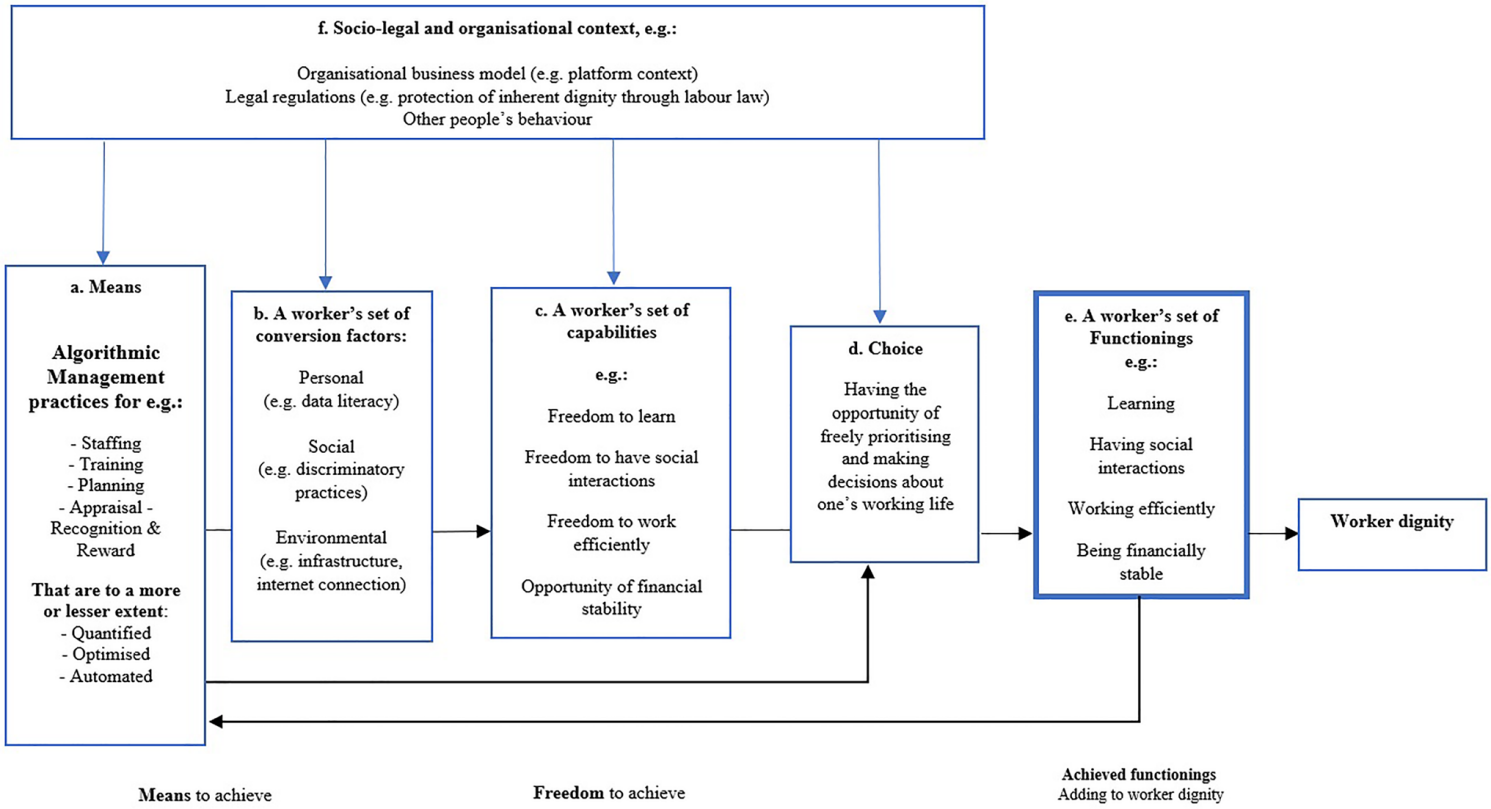
A schema of the relationships between key concepts in the conceptual model. **a** Means (box a) should be understood as the relevant (but not sufficient) conditions that allow capabilities to be created (box c). In this framework, Algorithmic Management practices are considered to be resources that could enhance (or hinder) the development of capabilities in the working life. **b** What an individual worker does with the provided means depends on their individual conversion factors. The conversion factors listed in box b are the factors that a worker has and employs to convert AM-based means/resources into capabilities. How means are converted into capabilities (box c) thus differs for each worker. **c** When individual conversion factors allow for it, the use of means can help to build or develop a worker’s set of capabilities, (box c), which are freedoms a worker has in their working life. Without conversion factors, AM-based means/resources will not add to the development of capabilities. **d** Next, it depends on a worker’s choices and priorities whether their capabilities are turned into actual functionings (achieved beings and doings). The feedback loop in this framework reflects that a worker’s choices (box d) are, under AM, often directly influenced by nudging techniques etc*. that are part of AM systems (box a). However, the behaviour of workers is also fed back into the AM system. ***e** The worker’s set of functionings (box e) are the realised capabilities: the actual beings and doings of the worker, which are the result of all the previous factors, and which together constitute a working life that is worthy of living. This means that a realised functioning adds to an agents dignity. **f** Finally, this development should be seen in the context of, and impacted by, the contextual factors, which can be socio-legal and organisational (box f)

Here, we would note that the role of the human manager is often underrepresented in the AM debate. Scholars such as Newlands (2020) and Veen et al. (2019) have highlighted this tendency. In this paper, we assume automation comes in various degrees, and we are not categorically excluding human involvement in management (as might be concluded from definitions such as that of Lee et al. (2015)). Rather, our goal is to develop a conceptual framework for *algorithmic management tools* that can be used to study and evaluate both the protection of inherent dignity as well as the promotion of contingent dignity. As such, we abstract and focus on the algorithmic elements of AM systems in practice. This is not to say we disregard other, non-algorithmic, managerial efforts that have a role in the use of AM systems. Rather we see the human manager as having a role in shaping AM and consider the role of human management in our conceptual framework by acknowledging various degrees of automation (see Fig. 1).

The final characteristic of AM considered is *optimisation*. Optimising the management of workforces is tightly linked with the *automation* characteristic of AM and entails control and large-scale (soft-)surveillance to increase efficiency (Duggan et al., 2020; Schildt, 2017). Frequently mentioned examples of highly optimised actions are those by platform firms that engage in algorithmic matchmaking and price surging (e.g. Rosenblat & Stark, 2016). The automation of these HRM activities allows organisations to replace human managers while simultaneously ensuring that the ‘right’ worker is assigned to the ‘right’ task for the ‘right’ price. While this enables organisations to optimise desired HRM outcomes, researchers have questioned whether this holds for those subject to AM: the workers. Consequently, we now proceed by explaining how the three defining features of AM have implications for the dignity of workers who are managed by algorithmic HRM practices.

### Dehumanisation and instrumentalisation in the AM debate

A closer look at the literature shows that AM is seen to limit human dignity through the *dehumanisation* and *instrumentalisation* of workers. Here, dehumanisation equates to the objectification of human beings and denial of their human attributes (Haslam, 2006), whereas instrumentalisation refers to the use of humans as ‘merely means to an end’ (Bal, 2017; Bal et al., 2020). For example, according to Moore and Robinson (2016), quantification at work compels workers “to squeeze every last drop of labour-power from their bodies” (p. 2775), implying that quantification adds to the instrumentalisation of workers. Similarly, Newlands (2020) discusses how datafication dehumanises workers because the data input into HRM algorithms are proxies that do not capture the full lived experiences of workers. Moreover, several studies show that AM instrumentalises workers through automation and optimisation (Kellogg et al., 2020; Newlands, 2020; Veen et al., 2019). As an example, Veen et al. (2019) show how AM enables platform firms to exercise control over workers in an automated and efficient manner to extract economic value out of them. Kellogg et al. (2020) show that algorithms at work allow companies to capture surplus value from, and thus instrumentalise, workers when they automate control over workers through algorithmic direction (e.g. algorithms are opaque and create information asymmetries), algorithmic evaluation (e.g. an automated performance appraisal) and algorithmic disciplining (e.g. automated nudging/rewarding and sanctioning of worker behaviour).

The examples above show that AM risks dehumanising and instrumentalising workers and therefore warrants study from the perspective of human dignity. They also show that the current academic debate on the effects of AM on working life primarily centres around how AM inherently *restrains* or *violates* human dignity. Conversely, some scholars have recently pointed towards the dual effects that AM might have on worker outcomes (De Stefano, 2020; Meijerink & Bondarouk, 2021; Wood et al., 2019). As De Stefano (2020) illustrates, the use of algorithms in management can both enhance and restrain the quality of working life. Wood et al. (2019) similarly acknowledge that, apart from creating power inequalities and pressuring workers, working in a digital environment governed by algorithms may also grant high levels of flexibility, autonomy, task variety and complexity. Along similar lines, Meijerink and Bondarouk (2021) argue that AM simultaneously enables and restrains the autonomy of workers. Therefore, it is important that AM research looks at both the restraining and the enabling effects of algorithmic HRM practices on worker dignity. Combining these aspects, a key message from this paper is that a conceptual framework is needed that allows AM to be studied from a perspective of human dignity, and that this conceptual framework should not only address how automated HRM practices might have *restraining* effects on worker dignity but also how AM might *promote* worker dignity. Having argued for such a framework and justifying the need for dignity-focused AM research, we turn to the classical interpretations of dignity that are relevant for management research.

## Dignity interpretations for Management Research

Although thinking about dignity and work has a rich history going back to philosophers such as Marx and Weber (Bal, 2017), links to dignity in management research are rare. Relatively recently, scholars such as Bal (2017), Dierksmeier (2011) and Pirson et al. (2016) have drawn attention to the need for a humanistic paradigm in management scholarship. One of the main arguments they advance is that incorporating dignity, as a core value in management science, will have positive outcomes for workplace conditions (Pirson et al., 2016). Further they argue it could help management research contribute better to societal welfare conditions. Below we outline two prominent interpretations of dignity that are considered to be important for management research (Pirson et al., 2016). We also discuss to what extent these interpretations can be identified in the debate on AM.

The first interpretation of dignity, referred to as *inherent dignity*, concerns human dignity that is an “essential attribute of human beings” (Pirson et al., 2016, p. 466). This inherent interpretation of dignity is frequently linked to the intellectual heritage of Immanuel Kant (Bal, 2017; Pirson et al., 2016). Notably, this Kantian view on dignity is often connected to human rights and a rules-based approach to protecting human dignity in, or from, endangering situations. Fundamentally, it stresses that people should not be seen and treated as ‘mere means to an end’ but as ends in themselves, thereby respecting their humanity. Moreover, people should have the autonomy to make their own rules and decisions. In connecting this interpretation to management thinking, Pirson et al. (2016) stress that: “Inherent human dignity is most salient when vulnerabilities (physical, psychological, social, economic) call for protection (in the persons of employees, managers, customers, suppliers, and other human stakeholders).” (p. 466). This suggests that management should protect workers from being instrumentalised, and protect worker autonomy to foster inherent dignity at work.

Considering the literature on AM, and how AM is seen as a problem for its dehumanising and instrumentalising effects on workers, it becomes apparent that AM is predominantly viewed through a lens of inherent dignity (and violations thereof). For example, scholars such as Moore and Robinson (2016), who criticise AM for its exploitation and instrumentalisation effects, are calling for the protection of the inherent dignity of workers. De Stefano (2020) explicitly argues for the protection of workers’ human dignity under AM through labour law, highlighting the value of a human-rights-based approach to labour regulation. Furthermore, in calling for a stop on instrumentalising workers, scholars such as Kellogg et al. (2020), Newlands (2020) and Veen et al. (2019) are, in fact, asking to end seeing workers as ‘a mere means to an end’. Overall, by highlighting the vulnerable positions of workers under AM, the current academic debate on AM is implicitly advocating the need to prevent the violation of the inherent dignity of workers subject to AM.

Second, there is an interpretation that reflects *contingent dignity*, which sees human dignity as an attribute that one can earn through actions. This view of dignity, as ‘something that can be earned’, is most commonly associated with Aristotelean virtue ethics (Pirson et al., 2016). Although it holds that every person can, in principle, have a dignified life, it is then up to the individual whether they realise this possibility. In this sense, contingent dignity differs from the inherent interpretation since that argues for the universal protection of all people, regardless of their preferences in life. Prison et al. (2016) stress that, in the management context, “contingent dignity is most salient when the self-esteem or self-respect of persons in a business context need to be promoted” (p. 466). To our knowledge, AM research is yet to explore the possibilities of algorithm-enabled HRM activities to help promote contingent dignity. A related study by Gal et al. (2020) applies a virtue ethics lens to study the impact of AM on workers. In so doing, they argue that AM, through datafication, nudging and creating information asymmetries, limits workers’ potential to flourish and cultivate their virtue. Accordingly, they propose rule-based solutions (e.g. algorithmic accountability, human oversight, limiting algorithmic nudging, counteracting reductionism) to avoid the negative impact of AM on workers’ contingent dignity. Although important, we believe this current account of contingent dignity under AM fails to consider how AM *promotes* (rather than violates) human dignity in an Aristotelean sense, and thus how workers, as active agents, can create a worthy life in work contexts where algorithms are deployed for HRM purposes.

In the current debate on AM, the inherent dignity interpretation dominates. This perspective leads to a focus on protecting workers from infringements of their dignity and encourages AM research to study the downsides of AM for workers through this lens (e.g. De Stefano, 2020). However, this interpretation tells us little about how AM might promote dignity by enabling humans to flourish (e.g. Gal et al., 2020). This is where the contingent (Aristotelian) interpretation of dignity comes into play. Another strength of viewing the Kantian idea of inherent dignity and the Aristotelian idea of contingent dignity as complementary is that it allows us to see how the responsibility for a dignified working life is actually shared between workers, employers and other stakeholders. Whereas, in a Kantian view, workers are often seen as people who need protection, adding the contingent dignity perspective gives workers agency to actively shape their own working lives. As such, our intention is to broaden the debate on AM by proposing a conceptual framework that integrates both interpretations of dignity. 

## A Capability Approach to Algorithmic Management

To understand how AM might violate *inherent dignity* as well as promote *contingent dignity,* we propose a Capability Approach (CA) as an alternative lens on worker dignity under AM. Over the past two decades, the CA, for which Amartya Sen (e.g. 1992; 1999) and Martha Nussbaum (e.g. 2000; 2006; 2011) laid the cornerstones, has been applied in many fields of study. It is most prominently used in political philosophy and development studies, but the theory has also been applied in welfare economics and social policy (Robeyns, 2005). Although the approach was first developed for macro-level development debates, it has increasingly found its way to applications on the micro-level. For example, Oosterlaken (2012) discussed the CA in relation to design, and authors such as Zheng and Stahl (2011) and Coeckelbergh (2011) considered the CA in combination with Information Technology. The argument we advance in this paper is that the CA offers a valuable basis for a conceptual framework for *worker* dignity – a micro-level phenomenon to which the CA has only been applied to a limited extent (Bertland, 2009; Cini & Goldmann, 2020). Further, the CA has not been widely used in the context of management. The main reason for this is that management scholarship has only recently (e.g. Pirson et al., 2016) and not yet extensively considered human dignity as an important value. This is despite, as we have illustrated, there being a need for dignity-driven AM research and the CA appearing a promising starting point.

Before presenting our framework and explaining how the CA helps to see AM as possibly both violating and promoting worker dignity, we outline the relationship between the two interpretations of dignity and the CA. In essence, the CA is a normative framework that acknowledges both the inherent dignity and contingent dignity concepts, while translating human dignity into tangible capabilities. According to the CA, what should be evaluated when looking at human development is not income, nor resources, primary goods, utility or preference satisfaction. Rather, the CA advocates a focus on *human capabilities and functionings*. Capabilities in this context are the positive freedoms or opportunities that people have and can choose to realise in what they view as valuable functionings (Robeyns, 2005). According to Sen, functionings are what people can actually achieve in terms of ‘beings and doings’, which together constitute what makes a life valuable (Alkire, 2005; Robeyns, 2005). The latter provides many examples of functionings, such as working, resting, being healthy, being recognised and being confident. As such, there is a distinction between achieved functionings and capabilities. Robeyns (2005) explains that this difference “is between the realised and the effectively possible; in other words, between achievements on the one hand, and freedoms or valuable options from which one can choose on the other.” (p. 95). That is, while capabilities reflect the opportunities that the individual has for living a worthy life, functionings manifest when the individual actually makes use of these opportunities and thus lives a life they find worthy of living. Summing up, the linkage between capabilities, functionings and dignity lies in the fact that capabilities are the necessary freedoms to achieve certain beings and doings: i.e., functionings. In turn, an increase in achieved functionings leads to an increase in an agent’s dignity (e.g. Coeckelbergh, 2011; Sharkey, 2014).

When we look at the fundamentals of the CA and the inherent and contingent perspectives of dignity, we can see that the CA respects both interpretations. First, the CA is universal in the sense that it can be applied to all human beings, regardless of their rational and physical capacities (Coeckelbergh, 2011; Nussbaum, 2006; Sharkey, 2014). Here we can see how the *inherent* dignity interpretation has a place in the CA: the development of capabilities and thus the opportunity to live a dignified life is important for all human beings due to their ‘inherent worth’. The CA has also been connected to the rules- and rights-based approach to protecting inherent dignity. As Nussbaum (2006) argued: “Indeed the capabilities approach is, in my view, one species of a human rights approach, and human rights have often been linked in a similar way to the idea of human dignity”. Specifically, the CA acknowledges the importance of rights and conditions for agents to develop capabilities and live a life they see worth living (Sharkey, 2014). Furthermore, the CA does not ignore factors that might limit the development of capabilities and thereby an agent’s dignity (Robeyns, 2005). Moreover, it does not ignore possible violations of a human’s inherent worth. From a managerial perspective, the CA allows one to determine when worker dignity should be protected in a management setting through rules, rights and contextual factors. For example, when workers in vulnerable positions should be protected against exploitation, discrimination or disrespectful interactions at work.

Second, although the CA is universal and incorporates the importance of protecting the inherent human value, it also responds to the preferences and opportunities of individuals. Here, it differs from the Kantian rules- and rights-based approach to dignity, which has universal rules for all agents. Thinking in terms of rules and rights leads to a ‘one size fits all’ perspective that does not easily allow one to take into account the individual circumstances of agents, their preferences and their interpretation of a good life. Moreover, it leads to a view in which workers are only subject to factors in their working life, and have no active role in shaping their own dignity. In contrast, the CA focuses on individual perceptions of a life worthy of living and the individual opportunities that are necessary to achieve it.

Through this individualistic aspect, the CA incorporates the idea of *contingent* dignity. With clear roots in virtue ethics as advocated by Aristotle (Bertland, 2009; Robeyns, 2005), the CA is an agent-based approach that can take account of individual-level the differences. These differences may occur both when individuals differ in their set of capabilities as well as when individuals choose to (not) make use of these capabilities, and thus translate capabilities into achieved functionings. In the specific context of workers under algorithmic HRM practices, such an agent-based account helps to accommodate the heterogeneity among the workers interacting with AM systems. Moreover, we argue that only looking at protecting the dignity of human beings through universal rules and rights limits the AM debate by highlighting only precarious situations in which workers need protection, whereas there are indications in the literature that, for some workers, AM can help build, rather than restrain, opportunities.

## Towards a conceptual framework

The remainder of this paper proposes a CA-inspired conceptual framework on worker dignity under AM as presented in Fig. 1. We discuss how, under AM, the various elements of this framework are relevant to worker dignity.^2^ This conceptual framework provides a lens to see whether worker dignity can be promoted and/or violated by AM, and how this could be acted upon. Although our conceptual framework is strongly based on earlier work by CA scholars and in particular the schematic representation by Robeyns (2005), we decided to adjust and extend the original CA framework to be able to better study dignity in a work-setting. The main difference between our framework and that of Robeyns (2005) is that we have transformed it in a way that makes it applicable to a micro-level work situation. This means, for example, that the social context represented by Robeyns (2005) involves in our framework also the organizational culture, and for example, the (platform) business model in place. Given that AM is used for managing workers/employees, we had to adapt the Robeyns (2005) framework further by excluding selected concepts like “goods” and “market production”, since these are not relevant conditions for an individual’s capability development at work. We have also added elements for the micro-level work context, such as the feedback loop that is discussed in the section on workers’ ‘choice’.

The schema (Fig. 1) provides an overview of key concepts in the CA (specific for AM), that is: the means, conversion factors, capabilities, choice, functionings and worker dignity, and how they are related (e.g. as conditions that need to be in place to enable a worker to convert means to capabilities, or choose to realise capabilities into achieved functionings that add to a worker’s dignity). The schema provides a representation of the conditions for capability development in a work context in which AM systems are deployed. It is a summary of the framework that is provided in the remainder of this article. This means that the Fig. 1. is not supposed to be read as a causal model. Rather, it is a conceptual tool that helps to understand what are the necessary and/or sufficient conditions for capability development and functionings in which a dignified working life manifests.

In line with the core focus of the CA, we start by discussing what a worker’s *capabilities* could be in their working life (box c). We proceed by positioning the AM practices within the framework. As explained below, we understand algorithm-enabled HRM practices as means that workers could use to influence their working life and develop capabilities. Therefore, we identify AM as *means* within the framework (box a). As means are relevant, but not sufficient conditions for capability development, next we consider what are necessary conditions to successfully develop capabilities out of means. These conditions are referred to as a worker’s *individual conversion factors* (box b) which – in combination with means – allow for capability development. The following step is to address the freedom of *choice* (box d) that workers have to turn their capabilities into *functionings* (box e), i.e., the achieved beings and doings that together make for a working life that is worth living. In addition, we consider how a worker’s choices and behaviours can in turn influence the AM systems they interact with (as suggested by the feedback loop in the framework). The final step is to discuss the *socio-legal and organisational context* in which the development of capabilities takes place (box f). In what follows, we discuss the key concepts that make up the CA and how these allow to see how AM can enable and restrain worker dignity.

### Capabilities

*Human capabilities* are central to the CA. Capabilities (box c) in this context are seen as opportunities for what agents are able to do and be at work. Whilst capabilities in life are often exemplified by the opportunity of being healthy, being able to make crucial decisions or having freedom of speech, these capabilities in life are different, and presumably more general, than capabilities in the *working* life. Consequently, some effort is required to translate and understand capabilities in a work context. Here, there is limited literature to draw on but a study by Abma et al. (2016) developed and tested a questionnaire, based on Sen’s account of the CA, to identify capabilities and how they were valued by workers. Capabilities in the workplace they identified include opportunities for learning, goal setting, decision-making, doing meaningful work and having an impact. Furthermore, Cini and Goldmann (2020) applied the CA to evaluate the situation of workers in Italian logistics and food delivery companies and found that workers invoke capabilities such as the opportunity to build a work-related identity, meet co-workers and resist managerial control.

However, to obtain a comprehensive impression of valued capabilities at work we need to extend our thinking. According to Robeyns (2005), the CA ultimately needs to be backed up by explanatory theories to be applicable to technologies or contexts.^3^ When seeking to identify worker capabilities, we can learn from the literature on dignity in the workplace since this explains how managerial practices help build capabilities at work. Examples of workers’ capabilities include the opportunity to do meaningful work, doing valuable work, earning a decent living, being proud, having status, being able to learn and develop skills, and the possibility to enjoy one’s work (Bal, 2017; Bolton, 2007; Hodson, 2001). Furthermore, we can consult existing studies on platform work (where AM is applied extensively) to explore what is valued by platform workers who have not been considered in previous capability research. Although AM is deployed increasingly beyond the platform economy, most examples of AM in the existing literature concern *platform* workers (Jarrahi et al., 2021; Meijerink & Bondarouk, 2021). This is why, in the remainder of this discussion, all the examples given concern workers in the platform economy. We would emphasise that the examples given are not exhaustive but are intended to illustrate how our framework, using elements of the CA, can help explain how worker dignity can be violated or promoted under AM.

On a general level, various studies highlight that the high level of flexibility, freedom and autonomy to decide when and where to work are common attractions of platform work and AM (e.g. Duggan et al., 2020). Zooming in on the question of why workers engage with platform work in the first place, Dunn (2020) provides valuable insights. He observes that motivations for engaging in platform work vary considerably, from escaping (other forms of) precarious or low-paid work to flexibility, the desire to have multiple jobs to generate supplementary income, interacting with others, or hedonism. Linking these findings to the development of capabilities can help identify what workers could see as opportunities developed through platform work under AM. For example, people who see platform work as a necessity due to their precarious financial situation, could hope that working under AM would strengthen their financial situation due to the flexibility in working hours. In this scenario, the worker capability that is being aimed for is the opportunity to achieve financial stability while at the same time being offered the freedom to engage in flexible work. Alternatively, some workers engage with platform work for social reasons (e.g. spending their leisure time as an Uber driver to meet new people) (Dunn, 2020; Möhlmann, 2015; Rosenblat, 2018). Capabilities that could be developed through AM for such a worker include meeting new people and storytelling. As such, two workers who are working with the same AM system, but engaging in platform work for different reasons, can have very distinct ideas of what platform work offers them and thus what ultimately may constitute worker dignity. Moreover, Dunn (2020) stresses that these motivations influence a worker’s perception of job quality, meaning that the reason why people engage with platform work can have implications for how they assess their job design and thus of being algorithmically managed.

The fact that there is such heterogeneity between platform workers has implications for our understanding of capabilities. As people working through platforms such as Uber do this for a vast variety of reasons (Dunn, 2020; Rosenblat, 2018), applying the same list of capabilities to them all would be a mistake. Within the literature on capabilities, some advocate a list of basic capabilities that are necessary for a dignified life (Sharkey, 2014).^4^ Although it is beyond the scope of this paper to engage in the prominent list debate, we briefly explain why we avoid using a pre-defined list of capabilities. We argue that what is needed for a dignified life cannot be decided by scholars and should remain in the hands of the workers themselves. Consequently, we do not wish to put together a list of basic capabilities as Nussbaum has advocated (Sharkey, 2014). The specific situation being studied, namely the use of AM, requires a dynamic approach that does not advocate universal rules or a strict set of capabilities for all workers. This dynamic approach also recognises that AM has many manifestations, that is, different organisations use software algorithms to automate and optimise different HRM activities. Moreover, it aligns with the broader aim of our paper, namely to include both the protection of *inherent* dignity (through rules, rights and freedom of choice) and the promotion of *contingent* dignity, which can best be assessed using an agent-based approach.

### Means

*Means* (box a) in the vocabulary of the CA are crucial for the development of capabilities. Here, means are all the material and non-material institutions and resources that allow capabilities to be created (Robeyns, 2005). Within the literature on capabilities and technology, “the usual way to define the relation between capabilities and technology is, as Sen did, to conceive of technology as one of the means to reach the aims (capabilities).” (Coeckelbergh, 2011, p. 84). In this paper, we build on existing studies which argue that AM – and HRM practices in general – are embedded with rules and resources (Meijerink & Bondarouk, 2018, 2021; Orlikowski & Scott, 2015), to argue that algorithm-enabled HRM practices, such as automated staffing, planning, training and appraisal, can be thought of as means for workers to develop capabilities. To understand how specific AM practices can, as means, shape opportunities for workers, we revisit the identified characteristics of AM: datafication/quantification, automation and optimisation. We now explain how these characteristics can both enable and restrain workers’ opportunities to live a dignified working life, and from this determine the use of AM as a means.

The first characteristic of AM that allows it to be a means for, as well as restraint to, capability development is *datafication or quantification*. It is argued elsewhere that the data-driven character of AM leads to negative effects for workers (e.g. Moore & Robinson, 2016) and therefore, limit workers to build capabilities. If we consider the use of *rating* systems in the automated HRM practice of *evaluation and appraisal* as an example, we see that several studies report that rating systems have a negative effect on workers when they are not allowed to explain bad ratings, or put them into context, while the platform regards these ratings as a leading indicator (Gandini, 2018). For example, on the platform Taskrabbit, workers that find and perform tasks are often recommended for further work based on their rating (Hannák et al., 2017). In such situations, the rating system leads to a fear of a bad rating and the possibility of being deactivated by the platform. Here, data-driven ratings do not amount to a means that positively contributes to a worker’s capabilities in terms of the opportunities in one’s working life (Hannák et al., 2017) and instead restrain capability development in terms of, for example, hindering the possibility to learn. Especially when considering those workers who engage in platform work as a necessity due to their precarious financial situation, one could argue that this practice does not help the development of capabilities but rather dehumanises workers and thereby endangers their inherent dignity. Conversely, some workers may use the automated rating system to the best of their ability and deploy it as an effective and convenient way of articulating their position in the market (Jarrahi and Sutherland, 2019). As an example, in a study of Uber taxi drivers, Cameron (2018) found that algorithm-generated performance ratings afforded them autonomy in terms of making choices through the work process to maximise earnings and/or reminded workers of the pleasant interactions they had had with passengers. Moreover, Lehdonvirta et al. (2019) show that algorithm-based performance ratings offer workers the possibility (and add to building capabilities) to charge higher fees for their services. In such cases, AM constitutes a mean that offers workers the capability (i.e., freedom/possibility) to generate income, experience meaningful work or be reminded of the impact they have on customers.

Algorithmic management also adds to – as well as limits – capability development through the *automation* of HRM activities. Here, the automated HRM practice of *training* can help illustrate how it again depends on the worker whether AM as a means actually helps to shape opportunities and adds to the capability set. As an example, Möhlmann and Zalmanson (2017) stress that AM, in terms of auto-generated predictions and recommendations focused on *training* and skills, could be of value to workers by providing, for example, optimised learning and talent development activities. Although this sounds desirable, it could be argued that part of a learning process is identifying skill gaps and individually deciding on specialisations and development. Conceivably, workers who engage in platform work due to their financially precarious situations will feel an urgency to gain the necessary skills to perform their tasks well and will want to be in charge of their own development process. Conversely, workers who engage with platform work for social reasons (e.g. wanting to meet new people) might care little about developing specific job-related skills. Again, this shows that whether a worker wants to develop and learn, and to what extent, depends on the personal interests of the worker and their views on a working life. As such, automated practices such as training and development recommendations can be a means that helps build a capability set, but not necessarily for all workers, and will contribute in various ways and to various extents.

Lastly, we turn to the AM characteristic of *optimisation* which allows seeing algorithm-enabled HRM as a means and hinderance for capability development. This characteristic can be seen to influence the effects of AM as a means in two ways. If we consider the example of *algorithmic matching,* some workers might well find the automated HRM practice of matchmaking helpful. For example, efficient matching could avoid taxi drivers having long, tedious waiting times between rides. Workers who especially engage with platform work for social reasons could benefit from this practice as it could help them gain more opportunities to meet new people. At the same time, other workers could find this optimisation practice restraining. Workers on platforms are often matched to the tasks they are good at (Kellogg et al., 2020) but remaining in a comfort zone and not extending to take on other tasks could also limit personal and professional growth. As was already argued for automated training, being able to learn on the job can be an important capability for workers who engage in platform work out of financial necessity, but an aspect where AM, in this context, does not help by providing a means for capability development. Consequently, it depends on a worker’s vision of what constitutes a good working life whether the matchmaking optimisation helps to shape opportunities, and thus promotes contingent dignity, or robs workers of the opportunity to learn and thus hinders their capability development. Nevertheless, the characteristics of AM strongly influence if and how AM can be seen as a means and, in turn, where AM is a means, it can both enable and restrain the development of human capabilities that workers consider essential.

### An individual worker’s set of conversion factors

Although algorithm-enabled HRM practices equate to a relevant means, in themselves they are not sufficient to create capabilities. The step from means to a capability set depends on an individual’s *conversion factors* (box b). That is, conversion factors help to convert algorithm-based means into capabilities. The rationale behind the individual conversion factors is that equality in terms of access to means does not lead to equal outcomes in terms of capabilities. People who are permitted to use certain technologies and services might be equal in terms of owning it, but not in their ability to use it properly and gain capabilities as a result. For this, they need conversion factors. This echoes insights from the HRM literature which show that the outcomes of a similar HRM practice differ across workers depending on the personal characteristics (e.g. knowledge, skills and abilities) they need to put such practices-as-means into use (Meijerink & Bondarouk, 2018). CA scholars posit that the conversion of a mean into a capability set hinges on three categories of conversion factors (Robeyns, 2005). We now outline the three types of conversion factors in the context of AM practices.

The first type, *personal* conversion factors, are characteristics of an individual that affect both bodily operation and psychological capacities. Under this category, Robeyns (2005) includes physical condition, reading skills and intelligence. In line with this, HRM research shows that a worker’s HRM competences (i.e., ability to engage with HRM practices) relate positively to the value that an HRM practice offers that worker (Meijerink et al., 2016). In terms of AM in the workplace, we therefore assume that important personal conversion factors relate to the level of understanding of managerial practices or data literacy (Jarrahi and Sutherland, 2019). If a worker does not understand how to optimally interact with the AM system, the algorithmic tools will be of little help in capability development (e.g. in working efficiently).

The second category, *social* conversion factors, is best understood in terms of social norms, discriminatory practices, gender practices, societal hierarchies, power relationships etc. which a worker can be affected by (Robeyns, 2005). An example of a social conversion factor would be being a member of a societal group that is affected by societal discriminatory practices (such as on basis of race and gender). Societal discriminatory practices like these have been transferred into AM systems through patterns of inequality in historical data (Kellogg et al., 2020). Several studies on the platform economy and AM report that groups of workers are systematically discriminated against by algorithmic software, for example by algorithmic rating leading to discriminatory outcomes (Greenwood et al., 2017; Kellogg et al., 2020; Rosenblat et al., 2017). As such, characteristics such as a worker’s gender, or membership of a societal group that is affected by other power-relations and societal hierarchies, can play a (restraining) role in converting an accessible means into a worker’s capability.

Finally, there are *environmental* conversion factors that include the provision of tangible, public goods, such as cycling lanes and street lighting, as well as intangible factors such as the climate, legislation and social infrastructure (Robeyns, 2005). In the context of AM, one could think about the accessibility of an effective internet or recent Covid-19 measures that have presented both opportunities and challenges to platform workers (Rani & Dhir, 2020; Spurk & Straub, 2020). Again, these factors influence how means are able to shape the capability sets of workers and thereby enable or restrain their opportunities in their working lives.

### From capabilities to functionings: choice

In themselves, worker capabilities do not equate to living a dignified working life. Rather, capability sets represent opportunities that people have to realise (what they view as) valuable ‘beings and doings’ (Robeyns, 2005). Having converted means into a capability set, it then depends on a worker’s choices which opportunities to actually use/leverage for turning capabilities into achieved beings and doings (box d). The latter reflects what Robeyns (2005) calls functionings. In general, it is recognised that workers in the platform economy have several capabilities that allow them to work flexibly with a somewhat entrepreneurial character. As Meijerink and Bondarouk (2021) put it, platforms such as Uber Eats and Deliveroo “grant gig workers the autonomy to enact their job demands/responsibilities as they wish and to use whatever job resources they prefer. Both platform firms try to avoid giving instructions on ‘how’ gig workers should perform their job” (p. 17). This means that, in general, workers have the freedom to choose which capabilities they use while working (e.g. whether they want to use the capability/opportunity to decline or accept new algorithm-made matches with clients). However, at the same time, the AM literature reports on some specific elements that restrain workers’ choices. There are several ways in which platforms control their workforces (Meijerink et al., 2021), including the often problematised nudging techniques and penalties (e.g. Walker et al., 2021). Ridesharing platforms incentivise workers to be active in ‘surge periods’ that are usually unpopular or would not otherwise have attracted them (Kellogg et al., 2020; Walker et al., 2021). The use of algorithm-based nudges thus indirectly or unobtrusively change the way workers prioritise use of their opportunities, and thus may prevent them from making a choice to deploy their capabilities for realizing achieved functionings. Supported by techniques from behavioural economics that ‘nudge’ workers, the AM system lets workers believe that ‘their choice’ is indeed just that (Walker et al., 2021). This interference with free decision-making power strongly appeals to the Kantian idea of autonomy, especially as the impact on free choice can go unnoticed. As such, it can be argued that algorithm-enabled HRM practices directly interfere with workers’ decision making autonomy (Leicht-Deobald et al., 2019; Meijerink & Bondarouk, 2021) and thereby risk violating their inherent dignity by restraining them in turning capabilities into functionings.

One AM-specific novelty that we have added to earlier frameworks such as that by Robeyns (2005) is a feedback loop from ‘choice’ to ‘means’. This is because, besides being affected by AM, we expect a worker’s choices to also shape AM. That is, not only can AM influence a worker’s choices and behaviours, the worker’s choices and achieved functionings also influence algorithm-enabled HRM practices. Indeed, several AM studies suggest the need to incorporate this feedback loop since workers try to influence AM through their behaviours and choices (Meijerink & Bondarouk, 2021). For example, Newlands (2020) explains how workers sabotage software algorithms by feeding them misleading data, thereby changing the output of the AM and, in turn, its value as a means. Irani et al. (2013) reported on workers building on their data literacy by deploying online scripts that track their online workplaces to gain insights into the algorithmic systems. Similarly, Jarrahi and Sutherland observe that: “By providing different inputs to the platform's various data collection processes, workers can alter, observe and improve its output, manipulating various platform algorithms.” (2019, p. 584). So, not only is the interaction between the workers and the AM system crucial for the development of capabilities, workers also have ways to shape the value of AM as a means.

### The socio-legal and organisational context

Finally, the CA requires one to consider the wider context in which the interaction between workers and AM plays out. CA scholars note the important role that macro-level factors play in capability development (Robeyns, 2005). Unlike a worker’s individual conversion factors, which differ per individual, the socio-legal and organisational context are wider, more general external forces. For example, the legal regulations covering working with algorithms apply to all workers interacting with AM systems under the same legal system. The most evident example of the *legal* context is the General Data Protection Regulation (GDPR) and labour-law regulations to protect workers where AM constitutes a means. The question is whether, and to what extent, AM is accounted for in the current legal system (De Stefano, 2020) and what role it plays in (1) the design of AM-as-a-means for capability development, (2) the support of workers in acquiring relevant personal conversion factors, and (3) affording choice to workers that are subject to AM. Mapping and evaluating the contextual factors (box f) can help identify possibilities, for example through legal protection, to protect workers in terms of inherent dignity. Alongside the wider social-legal context, our CA-inspired conceptual framework highlights the *organisational* context since platform firms may differ in their business and governance models. While some are for-profit firms funded by venture capital, others are cooperatives run by the workers themselves (e.g. Scholz, 2016). On the basis that these different types of organisations will rely on algorithm-enabled HRM practices in different ways, treat workers in different ways and grant differing amounts of autonomy, we argue that future research would benefit from studying how the organisational context, in which AM is deployed, influences AM as a means, workers’ personal conversion factors and their choices in deploying their capability set.

## Conclusions

In this paper, we have developed a conceptual framework, inspired by the Capability Approach (CA), for the study and evaluation of the effects of Algorithmic Management (AM) on worker dignity (summarised in Fig. 1). The CA was chosen as a plausible theory for assessing worker dignity as it can be applied to the work context and allows the integration of the Kantian approach to inherent dignity and the Aristotelean notion of contingent dignity. We concluded that the CA would indeed allow future research to study and evaluate how, and under what conditions, algorithm-enabled HRM activities can both violate and promote the dignity of workers subjected to AM.

By proposing our conceptual framework, we make the following contributions to the literature. First, adopting worker dignity as a concept when studying AM adds an important new perspective to the literature on algorithmic management that offers the possibility to move beyond the instrumentalist and dehumanising paradigm that is prevalent in AM research. An important message from this conceptual study is that the CA can reveal how algorithm-enabled HRM practices, such as automated matching, training, appraisal and evaluation, can have enabling *and* restraining effects on a worker’s capability set and thereby dual effects on worker dignity. As such, we expand on the existing AM literature, which is mostly focused on violations of *inherent* dignity and its protection, by offering a conceptual framework that enables future studies to simultaneously examine how algorithm-enabled HRM practices promote the *contingent* dignity of workers.

Although the framework was initially developed by scholars such as Sen, Nussbaum and Robeyns for evaluative purposes, we believe that it can serve multiple purposes for academics, designers, managers and institutional players (e.g. policy makers, labour unions) alike. Specifically, we propose our framework can be used in three ways. First, the framework can help to *describe* a situation in which an AM system is deployed. This helps to build a representation of the elements having a role in a workers capability development under AM and the social and organisational context at hand. Second, the framework can help to *analyse* the situation at hand and analyse what the desired and undesired aspects are. As the CA inspired framework can support both empirical and normative studies (Coeckelbergh, 2011), both qualitative and quantitative methodologies could be used to build knowledge on worker capabilities in the context of AM. For example, studies into the means, conversion factors, capabilities, etc. in a specific AM context could provide more insights on specific worker capabilities and eventually allow for normative assessment. Lastly, the framework can help to *normatively evaluate* the use of AM in the specific situation and work towards *design* features / practical solutions that might enhance the situation.

Accordingly, this framework cannot only be used by scholars, but can also provide insights to managers on where and when they can protect and promote worker dignity when working with AM systems. Further, it can provide programmers with insights on how the AM systems they develop affect worker dignity. For individual workers, the framework could help them gain an understanding of the actions they can take to develop capabilities and build a dignified working life.

By adopting a CA, research could help AM contribute better to societal welfare conditions. Future empirical studies could help fill knowledge gaps about the relationships and factors that play a role in these capability–AM relations. For example, research could explore the capabilities that workers need and/or desire, and how AM could be designed to enable the development of these capabilities. By using the conceptual framework, researchers could appropriately address the heterogeneity among workers and adopt an agent-based and dynamic approach to AM and dignity.

Overall, we hope that our CA-inspired framework assists academics in studying the impact of AM on workers as well as practitioners (e.g. managers, software developers, regulators, policymakers) in developing, regulating and/or evaluating AM to both prevent inherent dignity violation and promote the contingent dignity of workers.

## Footnotes

1 Speaking of AM as a unified system would be misleading, overlooking the complexity and impact of specific AM practices. Individual organisations can have separate software algorithms for various managerial practices. This is why our aim is to develop a conceptual framework that can respond to these complexities and evaluate AM practices, rather than assessing AM and its impacts as a single system.

2 The examples given in the schema are not exhaustive. Future systematic research into AM and capabilities is necessary to gain a comprehensive insight into the interaction between workers and the AM system, and how capabilities are built under AM.

3 For example, Zheng and Stahl (2011) combined insights from critical theory with the Capability Approach in the context of ICT. Along similar lines, Birdsall (2011) discussed how ICT and CA research could interact more and become better aligned. For a more extensive overview of the CA and ICT, see Oosterlaken and Hoven (2011a).

4 It could be argued (e.g. Sharkey, 2014) that Nussbaum’s account (e.g. 2000; 2006; 2011) by providing a list of basic capabilities is more specific than that of Sen (e.g. 1992; 1999). Having such a list makes sense when thinking about dignity in a social setting (e.g. care) where we think about human dignity. However, here we are speaking of worker dignity, in which there will be heterogeneity among workers and their views on the working life. We argue that, even without establishing a list of basic capabilities, the CA provides a tangible way to conceptualise worker dignity – of connecting dignity and work – which goes beyond while still respecting the protection of inherent dignity.
